# Cell Culture in a Hyperbaric Chamber: A Research Model to Study the Effects of Hyperbarism (Hyperbaric Pressure) on Bone Cell Culture

**DOI:** 10.3390/cells14161287

**Published:** 2025-08-19

**Authors:** Alessia Mariano, Valerio Consalvi, Enrico Marchetti, Angelo Rodio, Anna Scotto d’Abusco, Luigi Fattorini

**Affiliations:** 1 Department of Physiology and Pharmacology, Sapienza University of Rome, 00185 Rome, Italy; alessia.mariano@uniroma1.it (A.M.); luigi.fattorini@uniroma1.it (L.F.); 2 Department of Biochemical Sciences, Sapienza University of Rome, P.le Aldo Moro, 5, 00185 Rome, Italy; valerio.consalvi@uniroma1.it; 3 INAIL, Department of Occupational Health, Monte Porzio Catone, 00078 Rome, Italy; e.marchetti@inail.it; 4 Department of Human Sciences, Society and Health, University of Cassino and Southern Lazio, 03043 Cassino, Italy; a.rodio@unicas.it

**Keywords:** bone metabolism, hyperbaric environment, hyperbaric chamber, osteopenia, osteonecrosis, inflammatory pathways

## Abstract

The hyperbaric environment, to which many categories of workers are exposed, can provoke injuries that can lead to various types of disorders. A major part of the studies aiming to explore the causes/effects leading to these injuries are conducted in vivo. In the present manuscript, we describe the effects on osteoblast cell cultures stressed in a hyperbaric purpose-built chamber, using an in vitro model to analyze the affected pathways. A hyperbaric chamber for cell cultures was constructed by adapting a pressurized test chamber originally designed for technical use. The MG-63 cell line and human primary osteoblasts were placed into this chamber at different atm and exposure times, at 37 °C. After treatment, the chamber was depressurized by performing controlled decompression stops. Then, the pro-inflammatory cytokines and bone tissue biomarker expression were analyzed. The stress conditions induced the overexpression of pro-inflammatory cytokines, such as IL-6, IL-1β, and TNF-α, along with reactive oxygen species release. Moreover, the alteration of bone tissue marker production was observed. In particular, the increase in Receptor Activator of NF-κB Ligand (RANKL) and the decrease in Osteoprotegerin (OPG) were detected. Further modulation was observed regarding other biomarkers, Alkaline phosphatase, Osteocalcin, Bone Morphogenetic Protein-2, and mainly Collagen type I, all of which were downregulated by treatment. Taken together, these findings account for certain illnesses, such as dysbaric osteonecrosis, diagnosed in workers exposed to a hyperbaric environment. Inflammation induced by this kind of stress affects several factors involved in bone tissue homeostasis, leading to bone injuries, which are among the typical disorders observed in divers.

## 1. Introduction

Exposure to hyperbaric environments for working or recreational diving could lead to several pathologies, due to the extreme conditions, mainly for the increased hydrostatic pressure of the inhaled breathing mixtures. This is particularly important for professional divers involved in offshore oil and gas pipe development, archeological research, or in rescue actions. In particular, the professional divers involved in offshore saturation diving procedures have notably helped in gaining information to improve safety and safeguard the health of this group of workers [1]. There are also other groups of hyperbaric workers, mainly those involved in fishing activities or in fish farming, who are equally exposed to hyperbaric conditions, though not in saturation conditions, and usually, in a less controlled working environment. In these latter categories, several health problems have been reported with considerable variability [2]. These pathologies have been linked to gas bubbles formed by insoluble gases upon decompression. However, the inconsistent detection of gas bubbles has also prompted investigations into the inflammatory pathways and the formation of extracellular vesicles [3]. In the case of a prolonged hyperbaric exposure and rapid decompression, this pathology is attributed to nitrogen bubbles entering the fatty marrow of long bones and leading to reduced blood flow and subsequently to osteonecrosis [4]. On the other hand, there is growing evidence about the alterations of hematological and serological parameters, as well as alterations in inflammatory biomarkers upon exposure to dysbarism, breathing compressed air, or other gas mixtures, either during recreational or professional diving or therapeutic exposure to oxygen [3,5,6,7,8,9]. Notably, these alterations have been related to the expression of inflammatory and regulatory genes of the immune system response in neutrophils [10], most likely induced by the increase in reactive oxygen species. In addition, in a genome-wide gene expression study in saturation divers, selected plasma biomarkers for inflammation, vascular and endothelial function, and fibrinolysis were compared to the expression of genes involved in oxygen transport and in the production of endogenous antioxidants, finding that the expression of these latter genes was either upregulated or downregulated [11,12]. Among the health consequences observed in workers and sport divers is dysbaric osteonecrosis (DON). DON is associated with decompression sickness (DCS), although not all studies have found a correlation between DON and incorrect decompression procedures [2,13]. It has been postulated that certain individual predisposing factors, unrelated to decompression errors, may lead to DON, independently of the embolic or compressive effects of nitrogen bubbles. DON clinical signs may appear several years after hyperbaric exposure, due to the release of vasoactive mediators that slowly act as a procoagulant, producing intravascular coagulation [4,14,15]. The pathophysiology of the disease is not yet fully understood, and other etiological factors may have been overlooked.

The aim of this study is to evaluate the consequences of exposure of cultured osteoblast cells to elevated atmospheric pressure, to explore pathways that could putatively be involved in bone pathologies, such as osteopenia and osteonecrosis, triggered in vivo by hyperbaric environments. Previous studies that aimed to understand the mechanisms underlying the hyperbaric stress injuries were conducted in vivo and mainly referred to the damage of vascular or endothelial functions. This work shows aspects of novelty compared to other publications regarding in vitro studies on hyperbaric topics. In particular, Xin et al. analyzed the effect of elevated pressure on Meningothelial cells, using a pressure increase of 30 mmHg, exploring the effects on cell proliferation and reactive oxygen species production [16]. Moreover, some systematic reviews and meta-analyses have investigated the clinical effectiveness of hyperbaric oxygen therapy (HBOT) [17,18,19]. Previous manuscripts have analyzed the effects of HBOT in an in vivo model, both on healthy volunteers and diabetic patients [7,20,21,22].

To the best of our knowledge, this manuscript is the first one analyzing the effect of pressurized air, which resembles the conditions to which the divers are exposed, directly on osteoblast cells with a focus on inflammatory, oxidative, and typical bone pathways. The very innovative aspect of this work lies in the use of pressurized air instead of HBOT, allowing us to distinguish its specific cellular effects from those related to oxidative stress caused by high O_2_ levels. This fundamental distinction provides novel insights into the cellular adaptations of bone tissue to hyperbaric environments typical of diving and represents a significant methodological and conceptual advancement compared to previous research.

## 2. Materials and Methods

### 2.1. Hyperbaric Chamber

**Figure 1:**
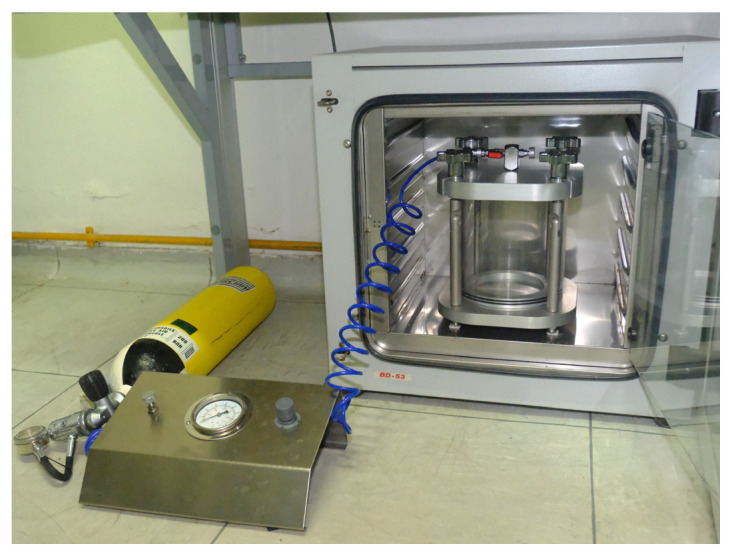
The hyperbaric chamber was constructed by PDE (Attrezzature Subacquee Professsionali, Genova, Italy) by adaptation of a pressurized test chamber for technical use.

The hyperbaric chamber was constructed by PDE (Attrezzature Subacquee Professsionali, Genova, Italy) through the adaptation of a pressurized test chamber for technical use. The square-shaped chamber (250 mm × 250 mm), made of polymethylacrylate (Figure 1) and aluminum, has been tested up to 10 atm. The chamber can be pressurized using a tank of compressed air connected via a low-pressure hose to a control console equipped with a manometer to indicate the pressure inside the chamber. The console can progressively increase the chamber pressure and has a drain tap to slowly release the pressure in the chamber at the end of the bottom time, simulating ascent. Decompression stops were always performed when required, and a 3 min stop at 3 m was consistently carried out for non-decompression dives. During the experiment, the chamber was disconnected from the console and was kept at 37 °C using an incubator (Figure 1). Control cells were kept under the same conditions but outside the pressurized chamber.

### 2.2. Cell Culture

Experiments were conducted both on the osteoblast cell line and on human primary osteoblasts; the cell line was chosen because it ensures good repeatability, while the primary cells are more suitable for studying pathways that could be altered in cell lines independently by treatment.

An osteosarcoma cell line, MG-63, was cultured in DMEM Glutamax (Gibco, Thermo Fisher Scientific, Waltham, MA, USA) supplemented with 10% fetal bovine serum (FBS), 1% penicillin/streptomycin, and 1% Na-pyruvate at 37 °C in 5% CO_2_.

Human primary osteoblasts (hOBs), from patients who underwent arthroplasty surgery, were isolated as previously described [23]. Full ethical consent was obtained from all donors, and the Research Ethics Committee, ASL Lazio 2 (#005605/2019, 3 March 2019) approved the study. Briefly, bones obtained from femoral and tibial condyles from patients who underwent a total knee arthroplasty were washed in sterile phosphate-buffered saline (PBS), minced, and treated with 1 mg/mL collagenase type IV plus 0.25% trypsin solution for 1 h at 37 °C under gentle agitation. After isolation, hOBs were grown to 80% confluence in McCoy’s medium (Sigma Aldrich, Co., Ltd., Saint Louis, MO, USA), supplemented with L-glutamine, penicillin/streptomycin, Fungizone, and 15% fetal bovine serum (FBS) (all purchased from Sigma Aldrich). The experiments were performed with cells at first passage (P1).

### 2.3. Treatment

Cells were plated in cell devices, cultured to 80% confluence, and then placed into a hyperbaric chamber at 2 and 4 atm for different times at 37 °C. As for the control, cells were placed at 37 °C in normobaric conditions (CTL). The experiments, both on the MG-63 osteosarcoma cell line and hOBs, were performed in Leibovitz’s L15 medium (GIBCO, Thermo Fisher Scientific) supplemented with required factors plus 1% FBS to overcome the problems that could arise from the absence of 5% CO_2_. This condition did not affect cell viability, both in the normobaric and hyperbaric environment.

After reaching the selected bottom time and 2 and 4 atm, the chamber was depressurized at a velocity corresponding to 10 m/min, and the required decompression stops were performed (Table 1), according to French decompression tables (https://www.legifrance.gouv.fr/eli/arrete/2019/5/14/MTRT1901236A/jo/texte, accessed on 11 April 2024).

After treatments, cells were prepared for the following analyses.

### 2.4. RNA Extraction and Retrotranscription

Total RNA from normobaric- and hyperbaric-cultured cells was extracted with Blood/Tissues Total RNA extraction kit (Fisher Molecular Biology, Trevose, PA, USA). The reverse transcription was performed according to the manufacturer’s instructions by Meridian Bioscience Reverse Transcriptase (Bioline reagent Ltd., London, UK).

### 2.5. RT-PCR

Quantitative Real-Time Polymerase Chain Reaction (RT-PCR) analysis was performed using an ABI Prism 7300 (Applied Biosystems, Thermo Fisher Scientific, Waltham, MA, USA). Amplification was carried out using SensimixPlus SYBR Master mix (Bioline). Primers (Table 2), synthesized by Bio-Fab research, were designed using Primer Express software v1.4.0 (Applied Biosystems). Data were analyzed by the 2^−ΔΔCt^ method, determining the transcript abundance relative to the 18S housekeeping gene.

### 2.6. Immunofluorescence

RANK and OSX proteins were visualized by immunofluorescence. Cells, plated at a density of 8 × 10^3^/cm^2^, were cultured in normobaric (CTL) and hyperbaric conditions for the required time. After treatment, cells were washed in phosphate-buffered saline (PBS), fixed in 4% paraformaldehyde in PBS for 15 min at 4 °C, and permeabilized with 0.5% Triton-X 100 in PBS for 10 min at room temperature (RT). Then, cells were blocked in 3% bovine serum albumin in PBS for 30 min at RT, and incubated for 1 h at RT with anti-RANK rabbit polyclonal primary antibody (bs-34045R, BIOS Antibodies, Woburn, MA, USA) diluted 1:300, and anti-OSX mouse monoclonal antibody (H00121340-M01, AbNova, Taipei City, Taiwan) diluted 1:50. Cells were washed with PBS and then incubated for 1 h at RT, or with Alexa Fluor 595 (red) donkey anti-mouse secondary antibody, or with Alexa Fluor 488 (green) goat anti-rabbit secondary antibody (Invitrogen, Thermo Fisher Scientific, Waltham, MA, USA) diluted 1:400. After washing, cells were stained with DAPI (Invitrogen, Thermo Fisher Scientific) to visualize the nuclei in blue. The images were captured by a Leica DM IL LED optical microscope, using an AF6000 modular microscope (Leica Microsystem, Milan, Italy). The densitometric analysis of protein production was performed using the free software ImageJ (Version 1.54p) (https://imagej.nih.gov/ij/, accessed on 18 August 2025).

### 2.7. ELISA

The amount of IL-6, IL-1β, TNF-α, BMP-2, ALP, and OC in the cell supernatant was determined using Enzyme-Linked Immunosorbent Assay kits (Fine Test ELISA, Fine Biotech Co., Ltd., Wuhan, China), whereas RANKL and OPG were measured using an ELISA test (Immunological Sciences, Rome, Italy) according to the manufacturer’s instructions. Optical Density (O.D.) absorbance was measured at 450 nm by a microplate reader (NeBiotech, Holden, MA, USA).

### 2.8. Superoxide and RNS Measurement

The intracellular superoxide and Reactive Nitrogen Species (RNS) have been measured by ROS-ID ROS/RNS Detection Kit (Enzo Life Sciences, New York, NY, USA) following the manufacturer’s instructions, using 5 × 10^4^/cm^2^ cells cultured in normobaric and hyperbaric conditions for 5 min and 25 min. The densitometric analysis of superoxide and RNS production was performed using the free software ImageJ (Version 1.54p) (https://imagej.nih.gov/ij/, accessed on 18 August 2025).

### 2.9. Statistical Analysis

All data were obtained from at least three independent experiments; each performed either in duplicate or in triplicate. Data were statistically analyzed with two-way repeated measures analysis of variance (ANOVA) with Bonferroni’s multiple comparison test, using Prism 5.0 software (GraphPad Software, San Diego, CA, USA). A *p*-value < 0.05 was considered significant.

## 3. Results

### 3.1. Inflammatory Pathway

**Figure 2:**
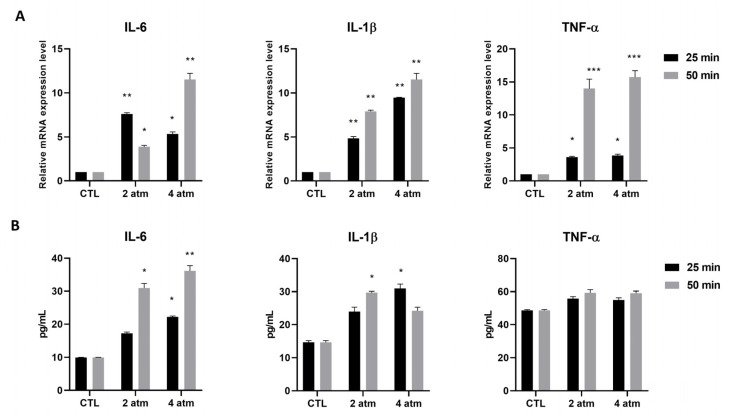
Effects of the hyperbaric environment on cytokine expression in the MG-63 cell line. Cells were cultured in a normobaric (CTL) or hyperbaric environment in the hyperbaric chamber at 2 and 4 atm for 25 and 50 min. After treatments, (**A**) mRNA was extracted and analyzed by RT-PCR. IL-6, IL-1β, and TNF-α mRNA levels were reported as relative mRNA expression levels with respect to 18S mRNA (2^−∆∆Ct^ method). (**B**) Cell supernatants were collected and analyzed by ELISA to determine the IL-6, IL-1β, and TNF-α amounts. The results are reported as pg/mL. Results are expressed as mean ± standard deviation (SD) of data obtained by three independent experiments. * *p* < 0.05, ** *p* < 0.01, *** *p* < 0.005 treated cell (hyperbaric conditions) vs. CTL (normobaric conditions).

MG-63 cells were subjected to 2 atm and 4 atm for 25 min and 50 min and decompressed, as described in Table 1. Subsequently, the mRNA expression of genes encoding for pro-inflammatory cytokines was analyzed. The results showed increased mRNA expression of IL-6, IL-1β, and TNF-α. IL-6 was increased when cells were subjected to both 2 atm and 4 atm, with 2 atm being more pronounced after 25 min, whereas 4 atm was more pronounced after 50 min compared to cells cultured in normobaric conditions (Figure 2A). The IL-1β mRNA increased at 2 atm and 4 atm at 25 min and 50 min, even if the increase was higher at 4 atm after 50 min exposure compared to cells cultured in normobaric conditions (Figure 2A). Finally, TNF-α was significantly higher after 50 min exposure compared to 25 min exposure, both at 2 atm and 4 atm relative to cells cultured in normobaric conditions (Figure 2A). The findings were confirmed when the amount of IL-6 and IL-1β proteins was measured, showing the same trend of the mRNA expression modulation (Figure 2B). Only IL-6 was higher at 4 atm compared to 2 atm, which represented the reversal of what was observed in the mRNA expression modulation. Moreover, the IL-1β protein level was not statistically increased after exposure at 2 atm for 25 min and at 4 atm for 50 min. Regarding the TNF-α protein level, the increase was not statistically significant at any pressure and time of exposure (Figure 2B). The same experiments were conducted on hOB cells, finding the same results after 25 min exposure (Supplementary Figure S1).

### 3.2. Oxygen and Nitrogen Reactive Species

**Figure 3:**
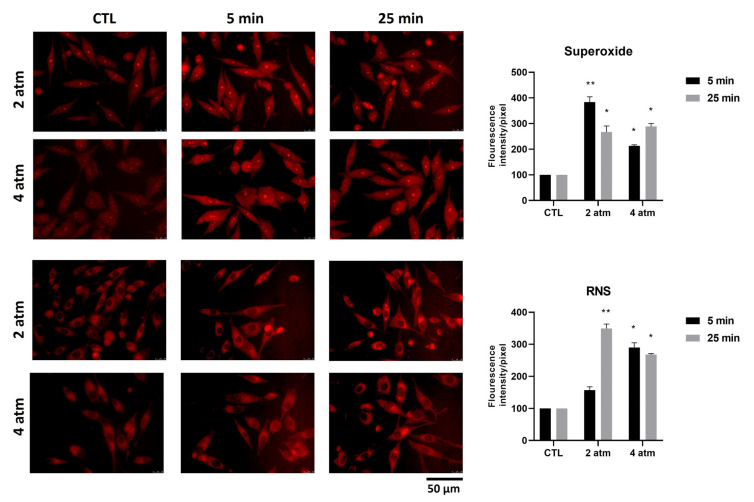
Effects of the hyperbaric environment on ROS and RNS production in the MG-63 cell line. Cells were cultured in a normobaric (CTL) or hyperbaric environment in the hyperbaric chamber at 2 and 4 atm for 5 and 25 min. After treatments, the amount of ROS and RNS produced was measured by ROS-ID ROS/RNS Detection Kit (original magnification 40×). The two upper panels show the superoxide species stained in red. The two lower panels show the RNS species stained in red. The histogram represents the pixel intensities in the region of interest, obtained by ImageJ. Results are expressed as mean ± standard deviation (SD) of data obtained by three independent experiments. * *p* < 0.05, ** *p* < 0.01 treated cell (hyperbaric conditions) vs. CTL (normobaric conditions).

Several types of stress induce cells to produce reactive oxygen and nitrogen species (ROS and RNS). To assess the production of ROS, in particular superoxide anion, and RNS, MG-63 cells were subjected to 2 atm and 4 atm for 5 min and 25 min, considering that these species are produced at early stages. The findings showed that the production of superoxide increased after 5 min exposure when subjected to 2 atm and after 25 min exposure when subjected to 4 atm (Figure 3, upper side). The RNS production, when the cells were subjected to 2 atm, increased after 25 min compared to 5 min, whereas they were increased to the same extent after 5 and 25 min when the cells were subjected to 4 atm (Figure 3, downside). Experiments on hOB cells showed almost the same results; the increase of ROS when cells were exposed to 2 atm and 4 atm for 5 min was not statistically significant (Supplementary Figure S2). Moreover, the increase of RNS when cells were exposed to 4 atm for 5 min was not statistically significant (Supplementary Figure S2).

### 3.3. Bone Remodeling Pathway

**Figure 4:**
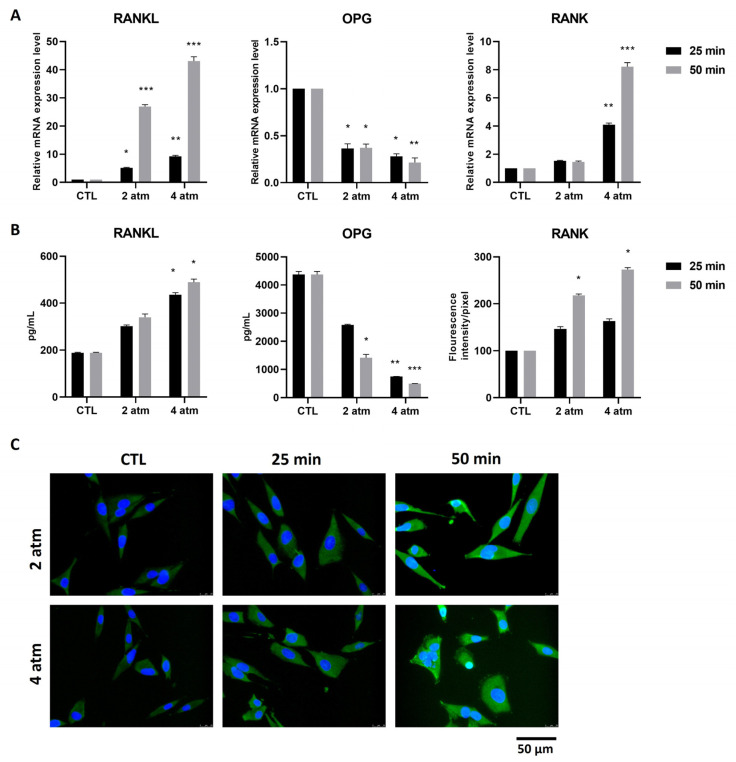
Effects of the hyperbaric environment on osteogenesis/osteoclastogenesis markers in the MG-63 cell line. Cells were cultured in a normobaric (CTL) or hyperbaric environment in the hyperbaric chamber at 2 and 4 atm for 25 and 50 min. After treatments, (**A**) mRNA was extracted and analyzed by RT-PCR. RANKL, OPG, and RANK mRNA levels were reported as relative mRNA expression levels with respect to 18S mRNA (2^−∆∆Ct^ method). (**B**) Cell supernatants were collected and analyzed by ELISA to determine the RANKL and OPG amounts. The results are reported as pg/mL. (**C**) RANK expression was analyzed by immunofluorescence using an anti-RANK primary antibody and Alexa Fluor 488 (green) secondary antibody. RANK is stained in green. Nuclei are stained with DAPI in blue (original magnification 40×). The histogram represents the pixel intensities in the region of interest, obtained by ImageJ. Results are expressed as mean ± standard deviation (SD) of data obtained by three independent experiments. * *p* < 0.05, ** *p* < 0.01, *** *p* < 0.005 treated cell (hyperbaric conditions) vs. CTL (normobaric conditions).

To evaluate whether the inflammatory pathways and the ROS/RNS production, activated by hyperbaric conditions, could be associated with bone formation/resorption signaling, we analyzed the RANK/RANKL/OPG pathway. MG-63 cells subjected to hyperbaric conditions, as stated above, showed that after 25 min and to a higher extent after 50 min exposure at 2 atm and 4 atm, produced a significant increase in Receptor Activator of NF-κB Ligand (RANKL) mRNA level (Figure 4A). At the protein level, a statistically significant increase was observed mainly after the exposure at 4 atm, both for 25 min and 50 min (Figure 4B). The Osteoprotegerin (OPG) is the antagonist of RANKL; the production of its mRNA, in our experimental conditions, was decreased after exposure at 2 atm and 4 atm for 25 min and 50 min (Figure 4A). A similar trend was obtained when the protein level was measured; the decrease was not statistically significant at 2 atm for 25 min (Figure 4B). The natural RANKL receptor is RANK, mainly present on the surface of osteoclasts. The interaction between the RANKL produced by osteoblasts and RANK present on the osteoclast membrane induces the activation of these latter cells. Moreover, the presence of RANK on the surface of bone marrow mesenchymal stem cells has been shown to represent an anti-osteoblastic signal [24]. We measured the production of RANK mRNA in MG-63 after exposure to hyperbaric conditions, finding that it was significantly increased after stimulation with 4 atm for both 25 min and 50 min (Figure 4A). At the protein level, the RANK expression was significantly increased both at 2 atm and 4 atm after 50 min exposure (Figure 4C). Considering that the presence of RANK in osteosarcoma cells has been described as a characteristic feature of these cells, we decided to continue the study on human primary osteoblasts (hOBs).

**Figure 5:**
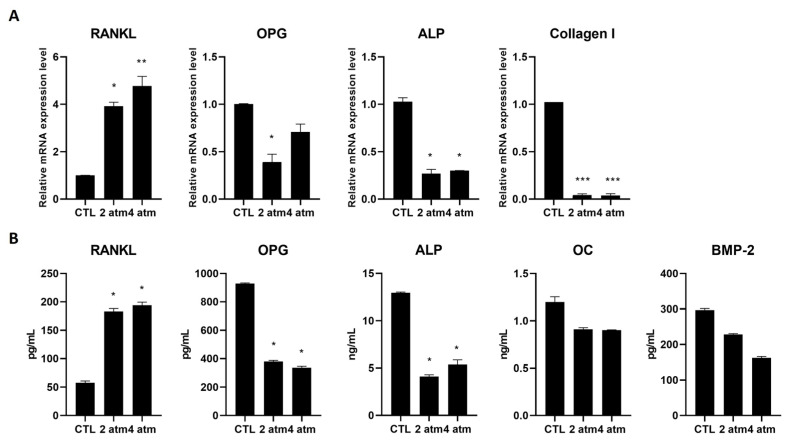
Effects of the hyperbaric environment on bone remodeling markers in hOB cells. Cells were cultured in a normobaric (CTL) or hyperbaric environment in the hyperbaric chamber at 2 and 4 atm for 25 min. After treatments, (**A**) mRNA was extracted and analyzed by RT-PCR. RANKL, OPG, ALP, and Collagen I mRNA levels were reported as relative mRNA expression levels with respect to 18S mRNA (2^−∆∆Ct^ method). (**B**) Cell supernatants were collected and analyzed by ELISA to determine the RANKL, OPG, ALP, OC, and BMP-2 amounts. The results are reported as pg/mL (RANKL, OPG, and BMP-2) or ng/mL (ALP and OC). Results are expressed as mean ± standard deviation (SD) of data obtained by three independent experiments. * *p* < 0.05, ** *p* < 0.01, *** *p* < 0.005 treated cell (hyperbaric conditions) vs. CTL (normobaric conditions).

The same conditions used to analyze MG-63 were applied to study hOBs, selecting a single exposure time, 25 min. The hOBs produced RANKL, both at the mRNA and protein level, at 2 atm and 4 atm for 25 min (Figure 5A,B). OPG decreased both at the mRNA and, to a greater extent, at the protein level (Figure 5A,B). Moreover, alkaline phosphatase (ALP) was measured both at the mRNA and protein level, finding that the hyperbaric conditions used induced a statistically significant decrease in this factor (Figure 5A,B). Collagen type I is the main type of collagen present in the bone extracellular matrix; thus, we analyzed its production and found that the exposure at 2 atm and 4 atm caused a marked decrease (Figure 5A). Finally, we measured the production of Osteocalcin (OC) and Bone Morphogenetic Protein-2 (BMP-2) at the protein level, finding both decreased by treatment, although not statistically significantly (Figure 5B).

**Figure 6:**
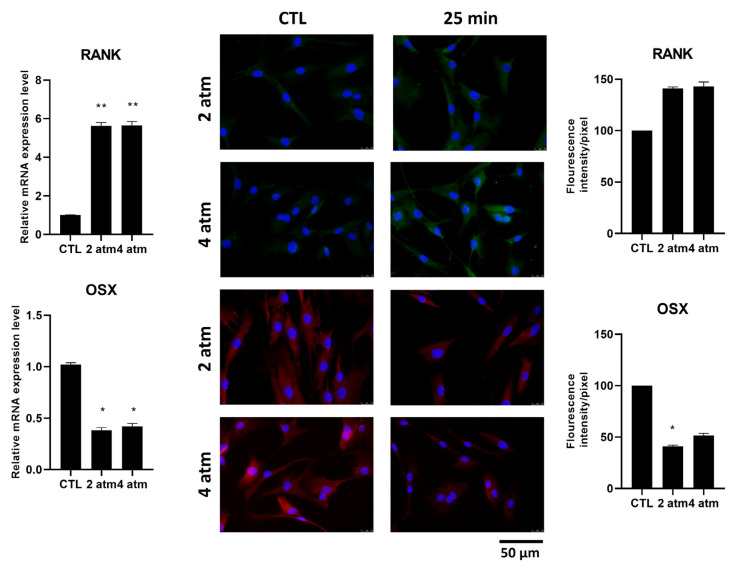
Effects of the hyperbaric environment on RANK and OSX markers in hOB cells. Cells were cultured in a normobaric (CTL) or hyperbaric environment in the hyperbaric chamber at 2 and 4 atm for 25 min. After treatments, mRNA was extracted and analyzed by RT-PCR. RANK and OSX mRNA levels were reported as relative mRNA expression levels with respect to 18S mRNA (2^−∆∆Ct^ method). RANK and OSX expression were analyzed by immunofluorescence using an anti-RANK primary antibody and Alexa Fluor 488 (green), upper panels, secondary antibody or anti-OSX primary antibody and Alexa Fluor 595 (red), lower panel, secondary antibody. Nuclei are stained with DAPI in blue (original magnification 40×). The histogram represents the pixel intensities in the region of interest, obtained by ImageJ. Results are expressed as mean ± standard deviation (SD) of data obtained by three independent experiments. * *p* < 0.05, ** *p* < 0.01 treated cell (hyperbaric conditions) vs. CTL (normobaric conditions).

The presence of RANK in mature osteoblasts is almost completely absent. We analyzed its production after exposure at 2 atm and 4 atm for 25 min. The mRNA levels were statistically increased, whereas at the protein level, they were not, and their localization was mainly in the cytoplasm (Figure 6, upper side). Among the transcription factors expressed in osteoblast cells, Osterix (OSX) is the transcription factor involved in the expression of some mature osteoblast genes, such as Collagen type I. We analyzed whether the exposure to hyperbaric conditions influenced its production and its localization, finding that both mRNA and protein levels were decreased after 25 min at 2 atm and 4 atm (Figure 6, downside). Moreover, in untreated cells, OSX was present both in the cytoplasm and in the nuclei. After treatment, the amount of OSX protein was generally decreased and was completely absent in the nuclei (Figure 6, immunofluorescent image, right downside).

## 4. Discussion

Many categories of workers are exposed to hyperbaric environments, which represent stressful conditions that can lead to several kinds of disorders. Divers, including those who are involved in offshore oil and gas pipeline development, archeological research, rescue operations, and fishing, are among the most affected by these injuries, often in the absence of decompression sickness (DCS). To understand the pathways affected by hyperbaric conditions, we studied the effects of elevated atmospheric pressure followed by decompression cycles on isolated osteoblast cells. The use of an in vitro system allows for more detailed investigation of signaling pathways. Moreover, the choice of bone cells was based on literature data that reported, among other symptoms, damage to bone tissue [2]. The early hematological and serological analyses recorded in hyperbaric environment workers, thus based on in vivo data, showed increased levels of pro-inflammatory cytokines and Reactive Oxygen and Nitrogen Species (ROS/RNS) [6,7,8,9,10,20,25]. To verify whether our experimental conditions, even when using an in vitro model and with pressurized air, could induce similar effects in osteoblast cells, we measured the release of IL-6, IL-1β, and TNF-α, finding that the level of all three cytokines was statistically significantly increased at all pressures and exposure times utilized. Only the TNF-α protein production as well as IL-6 protein production after exposure to 2 atm for 25 min did not statistically significantly increase, suggesting that a longer exposure may be required for protein production [26]. We could also hypothesize that the decompression would give cells the possibility to inhibit the transcription of TNF-α and IL-6 mRNA, limiting the damage. Of course, further studies are required to explore this hypothesis. Taken together, these results indicate that our in vitro experimental conditions were suitable to reproduce and study the alteration typically observed in workers exposed to hyperbaric environments. Consistently, ROS/RNS were altered too, further supporting the validity of this experimental approach. These findings were obtained using both the MG-63 osteosarcoma cell line and human primary osteoblasts (hOBs). Considering that we were primarily interested in understanding whether pathways typical of bone cells were altered by hyperbaric conditions, we analyzed the RANK/RANKL/OPG pathway. In healthy bone tissue, among other functions, the homeostasis between osteoblasts, involved in bone deposition, and osteoclasts, involved in bone resorption, is responsible for bone development and regeneration [27,28]. Some injuries can disturb this homeostasis, provoking bone degeneration and various bone disorders [29]. RANKL was initially described as being involved in the survival and proliferation of dendritic cells and T cells, and later it was found in bone marrow stromal cells as an osteoclastogenesis factor [24,30]. Nowadays, it is described as an osteoblast transmembrane protein, which is cleaved by metalloproteases and ADAMTS and released into the bloodstream, reaching the pre-osteoclast cells, which produce RANK. The interaction between RANK and RANKL stimulates osteoclast maturation, which in turn clears away the mineralized bone matrix. In healthy tissue, another protein, OPG, synthesized and released by osteoblast cells, participates in this pathway as a decoy receptor for RANKL, thus inhibiting the binding between RANK and RANKL, affecting the osteoclast stimulation [24]. Exposure of MG-63 cells to 2 and 4 atm pressure increased RANKL and decreased OPG, both at mRNA and protein levels, with the strongest effect at 4 atm, suggesting that this kind of exposure drives osteoblasts to stimulate osteoclast maturation and bone resorption. The effect is already evident in a short 25 min stimulus, and continues until 50 min, which is our longest time of exposure. RANK is expressed in several types of cells, such as dendritic cells and T cells, as well as osteoclasts [29]. Chen et al. showed that RANK is also expressed at an early stage of osteogenic differentiation of bone marrow mesenchymal stem cells, and it is downregulated after differentiation begins, due to its inhibitory effect on complete osteogenic differentiation [24]. We analyzed whether hyperbaric exposures could induce the RANK expression in MG-63 cells, finding that after exposure to 4 atm, the RANK mRNA was increased, whereas the RANK protein was increased both after 2 and 4 atm exposure. This result supports the findings regarding RANKL upregulation and OPG downregulation, further suggesting that hyperbaric conditions affect bone homeostasis, favoring osteoclast differentiation and affecting osteoblast metabolism concurrently. Although MG-63 cells are considered a good model to study osteoblasts, they are tumor-derived cells; therefore, several markers are abnormally upregulated in these cells. For this reason, we continued the experiments using hOBs stimulated at 2 atm and 4 atm for 25 min. Despite some limitations, hOBs can be considered a healthy model to study intracellular pathways. Notably, the RANK/RANKL/OPG pathway, as well as others, is not altered as it is in MG-63 cells. Considering that the effects on MG-63 cells were evident already after 25 min stimulation, we chose to use this time point while maintaining the two pressure conditions. Interestingly, we found that RANKL increased while OPG decreased. Moreover, RANK expression was significantly increased at the mRNA level and to a lesser extent at the protein level, confirming in hOBs the data obtained in MG-63 cells. Furthermore, we also analyzed the modulation of genes encoding for proteins typical of bone tissue, such as Alkaline Phosphatase (ALP), Osteocalcin (OC), Bone Morphogenetic Protein-2 (BMP-2), and Collagen type I. ALP is an early marker of osteogenesis, and its expression in vitro suggests the wellness of osteoblasts [31,32]. After hyperbaric stimulation, ALP was reduced both at the mRNA and protein levels. OC is another factor produced by osteoblasts, and it is involved in several bone metabolism pathways, such as bone matrix mineralization and hydroxyapatite crystal formation [32,33]. Moreover, decarboxylated OC exhibits endocrine activity controlling several physiological pathways [34]. Regarding BMP proteins, they have osteoinductive properties, thus promoting differentiation and maturation of osteoblasts [35,36]. We analyzed OC and BMP-2 protein secretion, finding that both decreased, although without statistical significance. Finally, Collagen type I, the most abundant collagen in bone, was dramatically reduced after hyperbaric treatment. To give an explanation to Coll I decrease, we checked the modulation of the Osterix (Osx, also called Sp7) transcription factor involved in osteoblast differentiation, maturation, and activity [37]. It belongs to the SP/KLF family, is mainly localized in the nucleus [38,39], and is involved in the expression of several bone proteins, such as Collagen type I a1, OC, and bone sialoproteins, all essential for osteoblast activity [39]. In our experimental conditions, Osx was decreased by hyperbaric exposure, both at the mRNA and at the protein level. Moreover, the residual OSX protein was not localized in the nucleus. An undetectable level of OSX production has been described in patients with both osteonecrosis and osteoarthritis [40]. Osteonecrosis, which in the past was suggested to be due to vascular impairment, has recently been suggested to be due to osteopenic bone, which in turn can be due to several etiological causes [41]. This recent evidence may suggest an interesting link between bone matrix loss and DON pathophysiology in subjects exposed to hyperbarism. Our hypothesis is that the inflammation produced by hyperbaric conditions affects the bone factors, which in turn affect the development and healthy maintenance of bone tissue.

## 5. Conclusions

The results reported in this manuscript clearly indicate that cultured bone cells exposed to a hyperbaric environment exhibit the same inflammatory alterations observed in hematological samples from workers exposed to similar conditions. The simple system of bone cells grown in vitro rules out any role as initial triggers of inflammation for the alteration in intravascular circulation or in the endothelial function, as well as any alteration of the immune response. These inflammatory alterations may be the result of exposure to oxygen and nitrogen at pressures above atmospheric levels, which induce the formation of reactive species of both gases within bone cells. Inflammation may subsequently lead to bone damage by decreasing morphogenetic protein levels and reducing collagen production.

Overall, these findings may shed light on the biochemical and molecular basis of osteonecrosis observed in many divers in the absence of decompression sickness. The decreased gene expression, and in turn protein production, of bone extracellular matrix components, due to the downregulation of transcription factors that control their expression, justifies the clinical signs of DON. To further investigate the effects of hyperbaric environments on cell culture, we are planning to explore additional pathways involved in bone homeostasis. Moreover, to better understand the impact of hyperbarism on workers chronically subjected to this stress, we are planning in vivo experiments in order to analyze the RANK/RANKL/OPG pathway in the serum of hyperbaric chamber operators, exposed to this stress daily.

## Figures and Tables

**Table 1:** French decompression table * for 5 min, 25 min, and 50 min of exposure at 2 and 4 atm.

Pressure	Time	1.2 atm	0.9 atm	0.6 atm	0.3 atm
2 atm	5 min				3 min
	25 min				3 min
	50 min				7 min
4 atm	5 min				3 min
	25 min			5 min	15 min
	50 min	3 min	10 min	25 min	45 min

* (https://www.legifrance.gouv.fr/eli/arrete/2019/5/14/MTRT1901236A/jo/texte, accessed on 11 April 2024).

**Table 2:** List of primers used for RT-PCR. The Accession Numbers are indicated.

Gene	Primer Sequences (Fw-Rv)
IL-6 NM_000600	5′-GATGGATGCTTCCAATCTG-3′ 5′-CTCTAGGTATACCTCAAACTCC-3′
IL-1β NM_000576	5′-ACGAATCTCCGACCACCACTA-3′ 5′-TCCATGGCCACAACAACTGA-3′
TNF-α NM_000594	5′-TCAGATCATCTTCTCGAACC-3′ 5′-CAGATAGATGGGCTCATACC-3′
RANK NM_003839	5′-CCTACGCACAAGGCGAAGATGC-3′ 5′-CGTAGACCACGATGATGTCGCC-3′
RANKL NM_033012	5′-TCAGCCTTTTGCTCATCTCACTAT-3′ 5′-CCAAGAGGACAGACTCACTTTATGG-3′
OPG NM_002546	5′-CGGCACATTGGACATGCTAA-3′ 5′-TCCCGGTAAGCTTTCCATCA-3′
Coll I NM_000088	5′-AAGGGTGAGACAGGCGAACA-3′ 5′-GACCCTGGAGGCCAGAGAA-3′
OSX NM_001173467.3	5′-AGAGCAACTGCTGGAGATC-3′ 5′-AAGCAGTGGTCTAGAGAGCC-3′
ALP NM_000478	5′-TGCGGAAGAACCCCAAAG-3′ 5′-ATGGTGCCCGTGGTCAAT-3′
18S NM_003286	5′-CGCCGCTAGAGGTGAAATTC-3′ 5′-CATTCTTGGCAAATGCTTTCG-3′

## Data Availability

Data are contained within the article and are available on request.

## Supplementary Materials

The following supporting information can be downloaded at https://www.mdpi.com/article/10.3390/cells14161287/s1, Figure S1: Effects of the hyperbaric environment on cytokine expression in hOB cells; Figure S2: Effects of the hyperbaric environment on ROS and RNS production in hOB cells.
