# Supplementary material for: Cell Culture in a Hyperbaric Chamber: A Research Model to Study the Effects of Hyperbarism (Hyperbaric Pressure) on Bone Cell Culture
Source: Cells. 2025 Aug 19;14(16):1287. doi: 10.3390/cells14161287 (PMC12385052; doi:10.3390/cells14161287)
Supplement: Supplementary file 1 [file cells-14-01287-s001.zip › cells-3754051-supplementary.pdf]

Supplementary Material

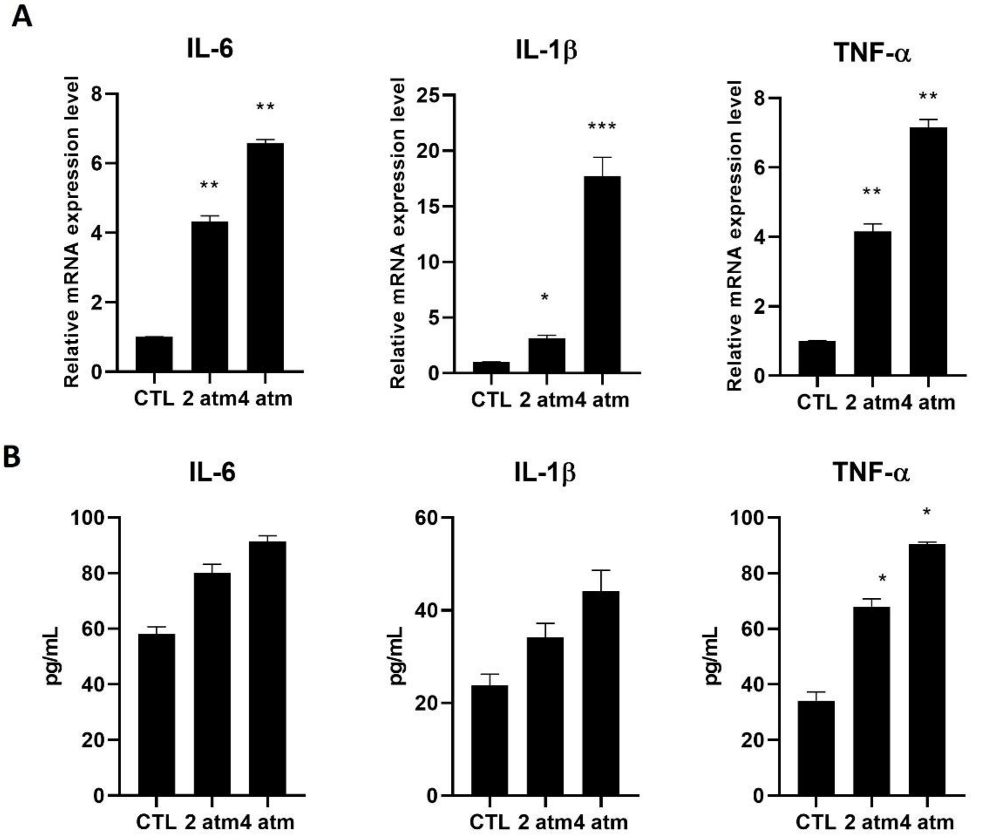

Figure S1. Effects of the hyperbaric environment on cytokine expression in hOB cells. Cells were cultured in normobaric (CTL) or hyperbaric environment in the hyperbaric chamber at 2 and 4 atm for 25. After treatments, **(A)** mRNA was extracted and analysed by RT-PCR. IL-6, IL-1β and TNF-α mRNA levels were reported as relative mRNA expression levels with respect to 18S mRNA ($2^{-\Delta\Delta Ct}$ method). **(B)** Cell supernatants were collected and analysed by ELISA in order to determine the IL-6, IL-1β and TNF-α amount. The results are reported as pg/mL Results are expressed as mean ± standard deviation (SD) of data obtained by three independent experiments. * $p < 0.05$, ** $p < 0.01$, *** $p < 0.005$ vs CTL.

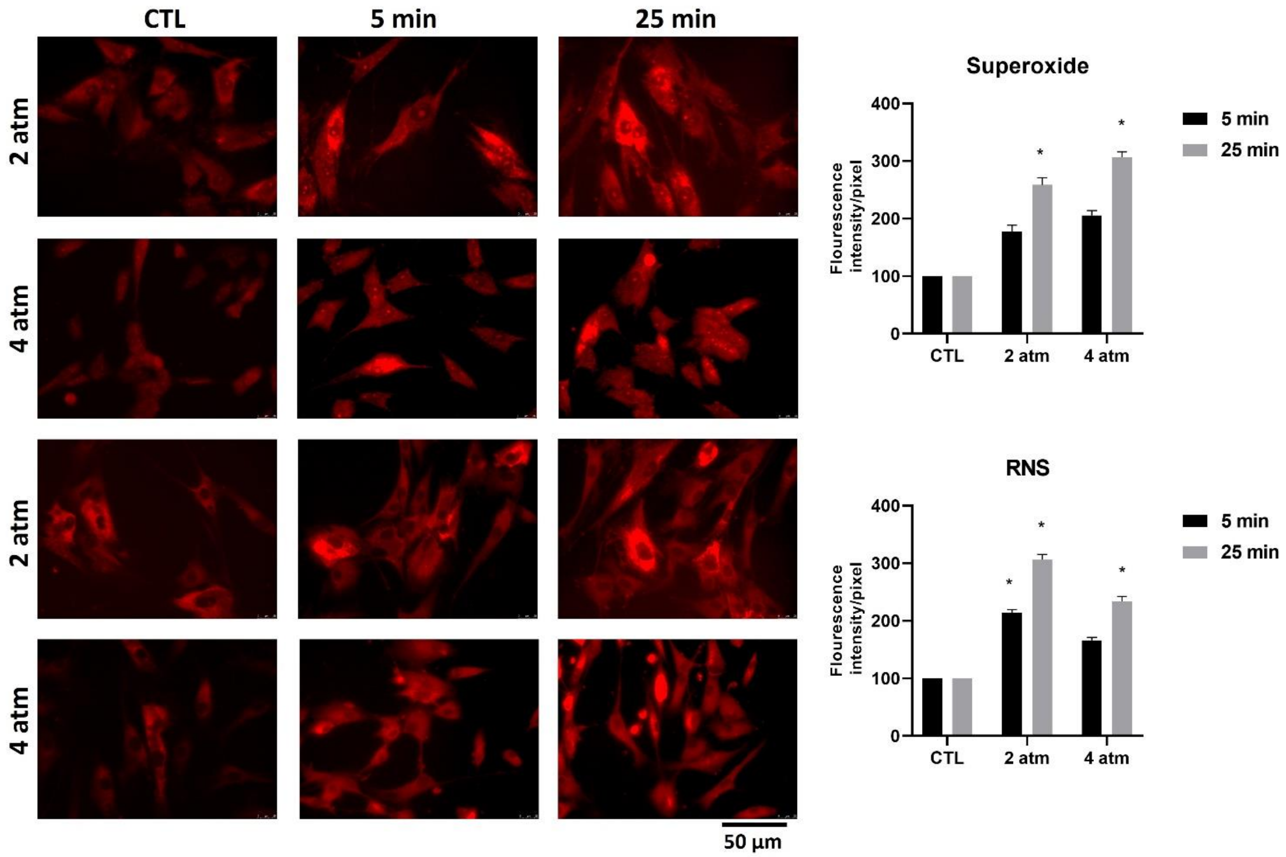

Figure S2. Effects of the hyperbaric environment on ROS and RNS production in hOB cells. Cells were cultured in normobaric (CTL) or hyperbaric environment in the hyperbaric chamber at 2 and 4 atm for 5 and 25 min. After treatments, the amount of ROS and RNS produced was measured by ROS-ID ROS/RNS Detection Kit (original magnification 40×). The histogram represents the pixel intensities in the region of interest, obtained by ImageJ. Results are expressed as mean ± standard deviation (SD) of data obtained by three independent experiments. * $p < 0.05$ vs CTL.
